# Supplementary material for: Small-molecule sensitization of RecBCD helicase–nuclease to a Chi hotspot-activated state
Source: Nucleic Acids Res. 2020 Jun 29;48(14):7973–80. doi: 10.1093/nar/gkaa534 (PMC7641324; doi:10.1093/nar/gkaa534)
Supplement: gkaa534_Supplemental_File [file gkaa534_supplemental_file.pdf]

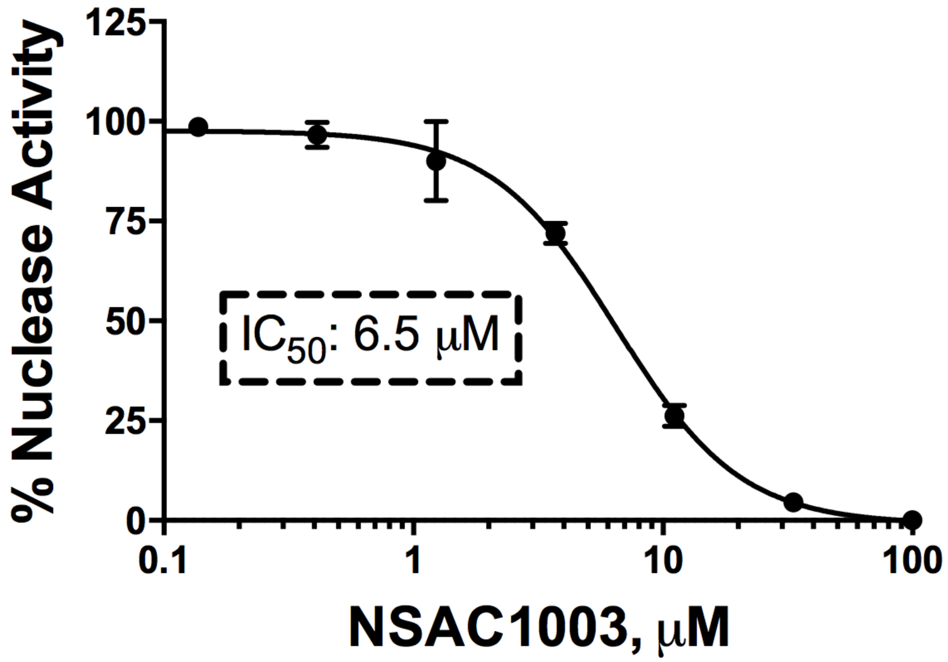

Figure S1. NSAC1003 inhibits RecBCD Chi-independent nuclease activity. Assays were done in the absence of Triton X-100 on two days. Filled circles are the means of the two experiments; individual data are at the error bars. In some cases the two experiments gave indistinguishable results, and the error bars are invisible. IC_{50} was 6.3 and 6.7 µM for the individual experiments. See also Figure 2.

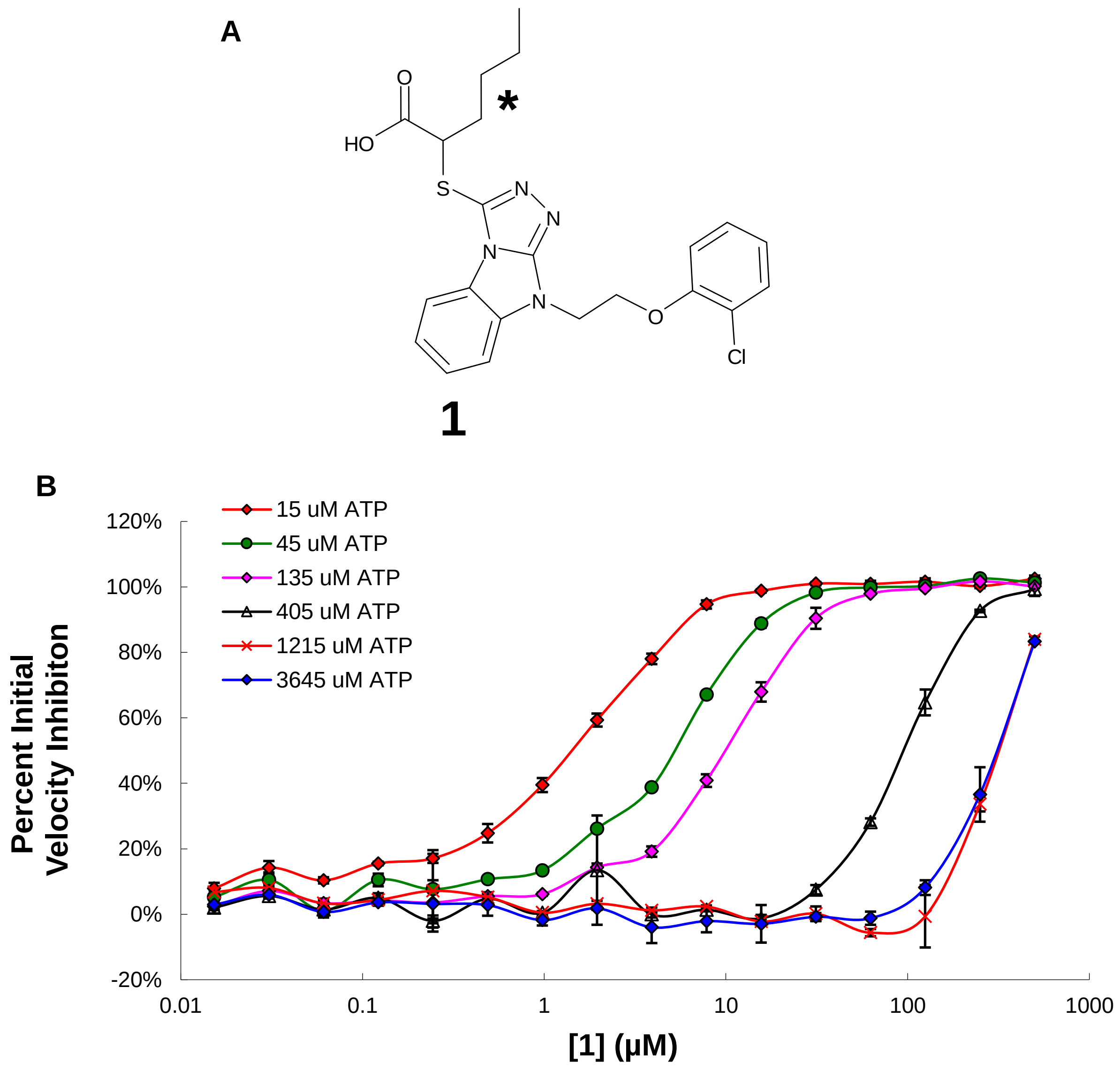

Figure S2. Compound **1**, closely related to NSAC1003, inhibits RecBCD Chi-independent nuclease activity in an ATP-competitive manner. **(A)** Structure of compound **1**, which differs slightly from NSAC1003 (Figure 2A): NSAC1003, a butanoic acid derivative, has an ethyl group where compound **1**, a hexanoic acid derivative, has a butyl group (asterisk). The IUPAC name is 2-({7-[2-(2-chlorophenoxy)ethyl]-2,4,5,7-tetraazatricyclo[6.4.0.0^{2},^{6}]dodeca-1(8),3,5,9,11-pentaen-3-yl}sulfanyl)hexanoic acid. **(B)** RecBCD nuclease activity was assayed in the presence of the indicated concentration of compound **1** and the indicated concentrations of ATP, using a PicoGreen dye-displacement assay (Tolun, G. and Myers, R.S. (2003) A real-time DNase assay (ReDA) based on PicoGreen fluorescence. *Nucleic Acids Res,* **31**, e111).

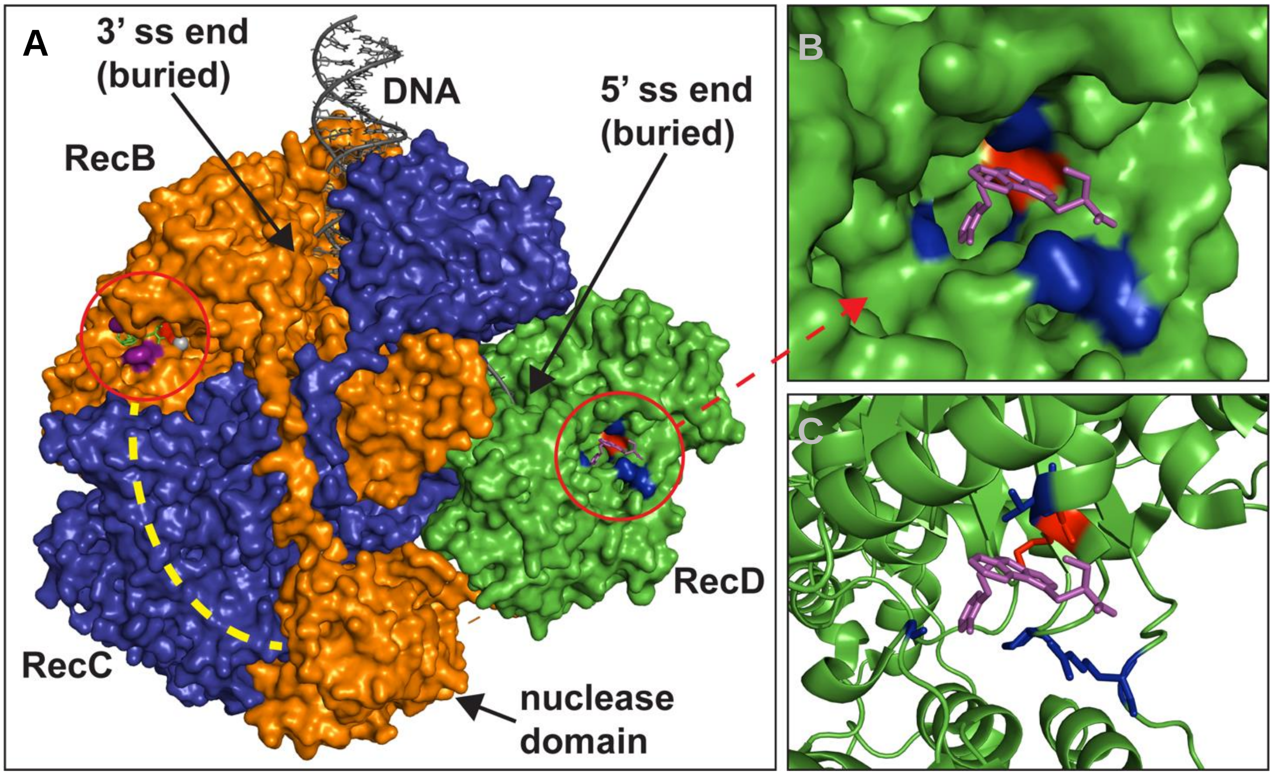

Figure S3. NSAC1003 docking to the RecD ATP site. **(A)** Using Docking Server (23), NSAC1003 (magenta sticks) was docked to RecBCD (surface view from PDB 5LD2 displayed as in Figure 3) at the ATP site in RecD (right red circle). A 20 Ǻ cube centered on the ATP-binding site was used for the calculation. Walker A box K177 (red) of RecD is shown, as are NSAC1003-interacting amino acids P173, T178, R360, and G543 (blue) of RecD. **(B)** Expanded view of part of panel A. **(C)** As in panel B with RecD as cartoon view except for K177 and NSAC1003-interacting amino acids P173, T178, R360, and G543 as sticks. See also Figure 3.
